# Pregnancy diagnosis based on pregnancy-associated glycoproteins detection in dairy cow milk: Factors influencing measurement

**DOI:** 10.1016/j.vas.2026.100672

**Published:** 2026-04-24

**Authors:** G. Bramante, L. Forte, O.M. Andriulo, A. Carbonari, A. Maggiolino, P. De Palo, A. D’Onghia, G.E. Dahl, V. Cicirelli, A. Rizzo

**Affiliations:** a Department of Veterinary Medicine, University of Bari Aldo Moro, S.P. per Casamassima km. 3 70010 Valenzano BA, Italy; b Associazione Regionale Allevatori Puglia, Strada Comunale San Nicola 2 70017 Putignano BA, Italy; c Department of Animal Sciences, University of Florida, Gainesville 32611, United States

**Keywords:** Pregnancy-associated glycoproteins, Pregnancy diagnosis, Dairy cattle, Gestational age, Milk composition, PAGs, Pregnancy Associated Glycoproteins, AI, Artificial Insemination, DIM_ Days in milk, SCS, Somatic cell score

## Abstract

Milk pregnancy-associated glycoproteins (PAGs) provide a noninvasive approach to early pregnancy diagnosis in dairy cattle. We retrospectively analyzed records from 2460 cows (Holstein and Brown Swiss) retrieved from a national livestock database (January 2022–April 2024) to identify cow-level and production factors associated with milk PAGs concentrations. Cows were categorized as pregnant, recheck, or not pregnant according to milk ELISA results, applying the threshold values provided by the manufacturer; outcomes were verified from insemination and subsequent calving data. Mixed models evaluated effects of breed, gestational age, parity, and days in milk (DIM), and Pearson correlations quantified associations with milk composition. Milk PAGs testing allowed a clear and unambiguous discrimination among the three diagnostic categories. Among cows classified as pregnant, 12% did not calve subsequently, whereas none of the recheck cows calved, underscoring the need for confirmatory follow-up. Absolute PAGs concentrations increased with advancing gestation (p < 0,01) and were higher in multiparous than primiparous cows (p < 0,01); DIM showed little effect. Weak positive correlations were observed between PAGs and milk protein (p < 0,01), casein (p < 0,01), and urea (p < 0,01), whereas associations with other milk traits were negligible. Collectively, these results support milk PAGs measurement as a practical and sensitive aid to pregnancy detection while emphasizing integration with routine clinical monitoring (e.g., rectal palpation and ultrasonography). Understanding how breed, gestational stage, and parity influence milk PAGs may improve test interpretation and contribute to more efficient reproductive management and reduced losses in dairy herds.

## 1 Introduction

Early pregnancy diagnosis in cattle is a key tool for improving reproductive efficiency, reducing calving intervals, and enhancing herd management (LeBlanc, 2013; Carbonari et al., 2024). One of the methods used for early pregnancy detection is based on the measurement of Pregnancy-Associated Glycoproteins (PAGs) (Barbato et al., 2022). These molecules are glycoproteins with molecular weights ranging from 37 to 90 kDa and belong to the aspartic protease family. However, unlike most members of this enzyme family, PAGs are enzymatically inactive due to substitutions within their catalytic motifs (Touzard et al., 2013; Gajewski et al., 2014; Barbato et al., 2022).

In cattle, from 1991 to the present, 22 distinct PAGs have been identified and designated sequentially as boPAG-1 to boPAG-22 (Zoli et al., 1992; Gajewski et al., 2014). These molecules are produced by both mono- and binucleated trophoblast cells and are stored in intracytoplasmic granules (Wallace et al., 2015). Following fusion with the endometrial epithelium, the trophoblast cells release their secretory granules containing PAGs into the maternal stroma (Green et al., 2005; Wallace et al., 2015). These glycoproteins subsequently enter the maternal circulation through the dense capillary network present in that area (Green et al., 2005). The role of these molecules is not yet fully understood, but previous studies have demonstrated their luteotropic activity at the ovarian level and immunosuppressive activity at the uterine level, both of which are essential for the establishment and maintenance of pregnancy (Dosogne et al., 2005; Wooding et al., 2005; Barbato et al., 2022).

Several studies have demonstrated that PAGs can be detected in the maternal bloodstream as early as day 22 post-insemination (Giordano et al., 2012), making them potentially useful for early pregnancy diagnosis (Kaya et al., 2016; Reese et al., 2018; Durocher et al., 2022; Akköse et al., 2025). However, greater diagnostic reliability is achieved from day 28 post-insemination, when plasma PAGs concentrations become more stable and indicative of pregnancy status (Gajewski et al., 2008; Ricci et al., 2015; da Silva et al., 2016).

Nevertheless, the limited practicality of large-scale blood sampling has shifted interest among farmers toward the use of milk as an alternative matrix for PAGs detection in bovine pregnancy diagnosis (Barbato et al., 2022). Milk sampling is minimally stressful for the animal and easy to perform by the farmer during routine milking, from which an aliquot can be conveniently collected for testing.

The dynamics of milk PAGs concentrations throughout gestation remain controversial, with available studies reporting inconsistent results (Gajewski et al., 2008; Lawson et al., 2014; Krebs et al., 2021; Ask-Gullstrand et al., 2023). However, there is consensus that, starting as early as day 28 post-insemination, milk can serve as a valuable matrix for early pregnancy diagnosis based on PAGs detection (Gajewski et al., 2008; Ask-Gullstrand et al., 2023). Currently, an ELISA commercial kit is available for the determination of PAGs levels in milk (Idexx Milk Pregnancy Test, Idexx Laboratories, Westbrook, ME), offering a sensitivity of 97,5% and a specificity of 82,2% and an accuracy of 91,9%, from day 28 after artificial insemination (Safak et al., 2025). Only a limited number of studies, many of which were conducted on relatively small and potentially inadequate sample sizes, have investigated factors influencing PAGs concentrations. These studies have yielded inconsistent and sometimes conflicting results, likely due to the limited sample size and consequent reduced statistical power. Moreover, it remains unclear whether these factors affect PAGs levels in a way that compromises the diagnostic performance of the test or simply influence PAGs concentrations without impacting its practical applicability.

The aim of this study was to investigate the potential influence of bovine specific factors, including age and breed, as well as gestational age, days in milk (DIM) and milk composition parameters (protein, fat, urea, pH, casein, and somatic cell score), on the concentration of PAGs in bovine milk.

## 2 Materials and methods

This is a retrospective study based on data collected from the Livestock Environmental Opendata (LEO) database, a digital repository containing all information related to the Italian livestock sector. No additional handling or intervention was required for the animals included in the study.

### 2.1 Dataset management

The initial database comprised 3000 dairy cows from 14 farms that underwent pregnancy diagnosis through PAGs testing between January 2022 and April 2024. For each animal, data were extracted from the LEO database, including:

animal information: date of birth, breed, and number of lactations;
reproductive records: date of last insemination, previous calving date, date and number of calves born at calving related to the pregnancy under consideration;
PAGs analysis data: date of analysis, test result, and PAGs concentration. Based on the outcome of the PAGs analysis obtained from the database, animals were classified as "pregnant", "not pregnant" or "recheck”.

Milk parameter data: milk testing data recorded on the same day as the PAGs analysis were extracted in order to obtain values for specific milk parameters, including fat, protein, urea, pH, casein, and somatic cell count.

Starting from the initial database, exclusion criteria were applied: all crossbred cows and cows of breeds other than Brown Swiss and Friesian were excluded; cows with twin pregnancies were excluded; and all animals with missing data for any of the recorded variables were removed. After applying these criteria, the final dataset consisted of 2460 dairy cows. Subsequently gestational age was calculated by comparing the date of PAGs analysis with the date of the last insemination, while DIM were determined by comparing the PAGs analysis date with the date of the previous calving. Following this assessment, it was established that the animals in the database ranged between 28 and 180 days post-AI and between 80 and 450 DIM.

The "pregnant" cows were further stratified according to gestational age (days post AI) into four categories:

cows pregnant for <60 days;
cows pregnant for 61–90 days;
cows pregnant for 91–120 days;
cows pregnant for >120 days.

This stratification was adopted in accordance with the critical phases of bovine pregnancy proposed by Wiltbank et al., 2016, taking into account the physiological changes that occur during these phases (Wiltbank et al., 2016). These stages are characterised by dynamic changes in trophoblast activity, endocrine signalling and placental development (Schlafer et al., 2000; Wiltbank et al., 2016). All of these factors are known to influence pregnancy maintenance and the secretion of pregnancy-associated glycoproteins (Touzard et al., 2013).

Additionally, based on DIM, they were classified into:

cows with equal or <180 DIM;
cows with >180 DIM.

Finally, for animals categorized as “pregnant” and “recheck," the real pregnancy status was confirmed by evaluating insemination data and the occurrence of a subsequent calving following the PAGs analysis.

**Fig. 1:**
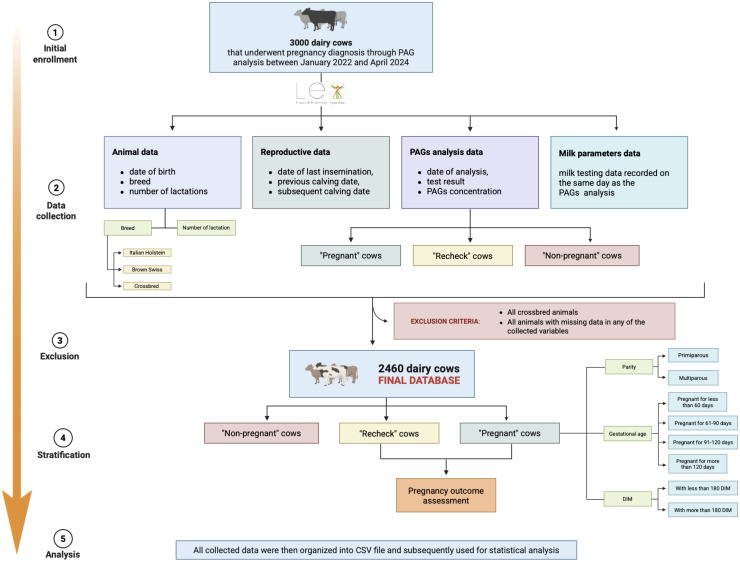
Workflow representation outlining the steps undertaken in the execution of this study.

All collected data were then organized into CSV file and subsequently used for statistical analysis. A schematic representation of the database management is provided in Fig. 1.

### 2.2 Milk parameters

Milk samples were collected by the Italian Breeder Association during routine milk recording activities conducted on dairy farms in the Apulia region. Milk was sampled from all four quarters after teat disinfection and discarding of the first streams of milk.

The following milk parameters were considered: fat (g/100 g), protein (g/100 g), urea (mg/dL), pH, casein (g/100 g), and somatic cell count (n × 1000/mL).

The analysis to obtain all these parameters were conducted by the Italian Breeder Association. These analyses were performed using Fourier-Transform Infrared Spectroscopy (FTIR) via the MilkoScan™7RM instrument (FOSS, Hillerød, Denmark), a high-performance infrared analyzer routinely used in dairy laboratories for standard milk component testing. The Milkoscan’s analytical principle is based on the absorption of mid-infrared radiation by specific molecular bonds within milk components. Each parameter exhibits a distinct absorbance pattern at a specific wavelength, allowing for simultaneous quantification of multiple constituents in a single measurement. Calibration curves were established and routinely verified according to ISO 9622:2013 standards (ISO9622/IDF141, 2013), ensuring traceability and consistency of the results. The MilkoScan7 RM provides following precision details: repeatability CV < 0.5 % for fat, protein, lactose, and solids; accuracy CV < 1.0 % in bulk samples and < 1.5 % for single-cow milk; urea measurement has standard deviation < 1.5 mg/dL, temperature repeatability < 1 m °C.

Somatic cell counts (SCC) in milk were determined using Fossomatic™ 7, a flow cytometry based instrument (FOSS, Hillerød, Denmark), compliant with ISO 13,366 2/IDF 148 2:2006. The instrument features a low working factor of 100 for enhanced repeatability. According to technical specifications: Repeatability (intra assay precision):

CV < 6 % for 100–299 ×10³ SCC/mL
CV < 4 % for 300–499 ×10³ SCC/mL
CV < 3 % for 500–1 500 ×10³ SCC/mL

Accuracy: relative mean difference to reference Direct Microscopic Somatic Cell Count (DMSCC) < 10 % and Carry over: < 1 % relative

Somatic cell counts, obtained separately via flow cytometry instrument (FOSS), were converted to Somatic Cell Score (SCS) using the logarithmic transformation formula:

$$\text{SCS}=\text{lo}g_{2}\left(\text{SCC}\;/100,000\right)+3,\;\text{as}\;\text{per}\;\text{standard}\;\text{dairy}\;\text{herd}\;\text{management}\;\text{protocols}.$$

### 2.3 PAGs assessment

PAGs levels were assessed using a commercial ELISA kit (IDEXX Laboratories Inc.) at the Apulian Regional Breeder Association in Putignano, Italy. Pregnancy status (pregnant, non-pregnant, or recheck) was determined based on the optical density of the sample, using the threshold values provided by the kit manufacturer. Milk PAGs ELISAs were performed in the same laboratory following the manufacturer’s ELISA protocol consistently processed by the same operators to minimize potential inter-laboratory variability. Microtiter plates were pre-coated with a monoclonal antibody specific to pregnancy-associated glycoproteins (PAGs). Diluted milk samples were added to the wells and incubated, allowing PAGs present in the samples to bind to the immobilized antibody. Detection was carried out using a PAG-specific antibody conjugated with horseradish peroxidase. Following a washing step to remove unbound conjugate, the chromogenic substrate 3,3′,5,5′-tetramethylbenzidine (TMB) was added. The intensity of color development, directly proportional to the concentration of PAGs in the sample, was measured spectrophotometrically. All ELISA measurements were performed in single runs according to the manufacturer’s instructions. Results were expressed as S-N values, calculated as the optical density (OD) of the sample (S), corrected by subtracting the reference wavelength OD, minus the OD of the negative control (N), also corrected for its reference wavelength, at 450 nm. Each microplate included both positive and negative controls.

Pregnancy status was determined based on threshold values established by the ELISA kit manufacturer. Specifically, cows with an S-N value < 0.100 were classified as “not pregnant”; those with values between > 0.100 and < 0.250 were categorized as “recheck”; and cows with S-N values ≥ 0.250 were classified as “pregnant”.

The anti-PAGs monoclonal antibody used in this test was produced against the PAG-55 protein fraction comprising PAG-4, PAG-6, PAG-9, PAG-16, PAG-18, and PAG-19 (Mathialagan et al., 2009). Although individual members of the PAGs family exhibit distinct temporal expression patterns throughout gestation, the broad antigenic affinity of the antibody to multiple PAGs isoforms allows for the detection of pregnancy-associated glycoproteins throughout the entire gestational period (Green et al., 2000; Mathialagan et al., 2009). Therefore, the ELISA assay used in this study is able to detect dynamic changes in PAGs expression during pregnancy, without being limited to a specific time window.

### 2.4 Statistical analysis

We performed descriptive analyses using Microsoft Excel (Microsoft Office 365; Microsoft Corp., Redmond, WA, USA). All other analyses were performed using the SAS software package (SAS, 2018). All data were first assessed for normality using the Shapiro–Wilk test. In addition, residuals from the mixed models were inspected to verify normality and homogeneity of variance before result interpretation. Subsequently, PAGs variables were analysed using general linear mixed models according to the following models:

(1) $$Y_{\text{ijk}}\;=\mu +\alpha_{i}+\sigma +I_{j}+\varepsilon_{\text{ijk}}$$

where Y_ijk_ rapresents the parameter as dependent variable, µ is the overall mean, α_i_ is the random effect of the i^th^ cow, σ is the covariate accounting for the seasons, I_j_ is the fixed effect of days post-artificial insemination (j = 1, …, 4), and ε_ijk_ is the error term;

(2) $$Y_{\text{ijk}}=\mu +\alpha_{i}+\beta +\sigma +P_{j}+\varepsilon_{\text{ijk}}$$

where Y_ijk_ rapresents the parameter as dependent variable, µ is the overall mean, α_i_ is the random effect of the i^th^ cow, β is the covariate accounting for DIM, σ is the covariate accounting for seasons, D_j_ is the fixed effect of parity (j = 1, 2), and ε_ijk_ is the error term. The results are expressed as least square means and standard error of the means. The significance level was set to *P* < 0.05. Moreover, all data were subjected to Pearson correlation analysis; Pearson’s r was reported as an estimate of effect size together with the corresponding two-tailed *P*-values are reported (α = 0.05). All statistical analyses were performed on the full dataset and separately within each breed (Brown Swiss and Holstein).

## 3 Results

### 3.1 Description of database

Starting from an initial database of 3000 animals, the application of the exclusion criteria resulted in a final database of 2460 dairy cows, of which 1386 were Holstein and 1074 were Brown Swiss. All animals were between 2 and 8 years of age and in their first to sixth lactation. In addition, based on the outcome of PAGs testing, the final dataset included 1750 cows classified as “pregnant”, 568 as “not pregnant”, and 142 as “recheck”.

**Fig. 2:**
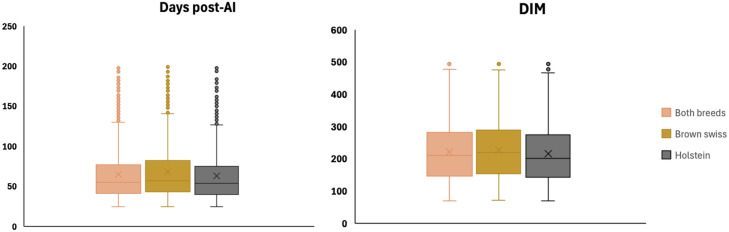
Distribution of cows in the dataset according to days post–artificial insemination (days post-AI; left) and days in milk (DIM; right). Boxplots display the median (horizontal line within each box) and mean value (cross inside each box). Results are reported for the overall population (Both breeds n = 2460), as well as separately for Brown Swiss (n = 1074) and Holstein cows (n = 1386).

**Fig. 3:**
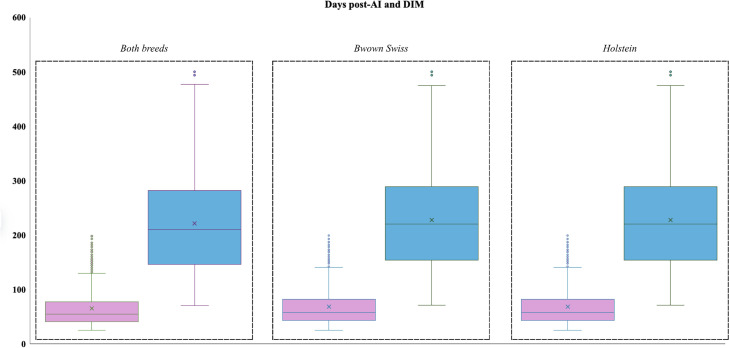
Boxplot representation of days post–artificial insemination (days post-AI, pink) and days in milk (DIM, blue) in the overall population (Both breeds n = 2460), Brown Swiss (n = 1074), and Holstein cows (n = 1386). For each distribution, the horizontal line inside the box denotes the median, while the cross indicates the mean value.

**Fig. 4:**
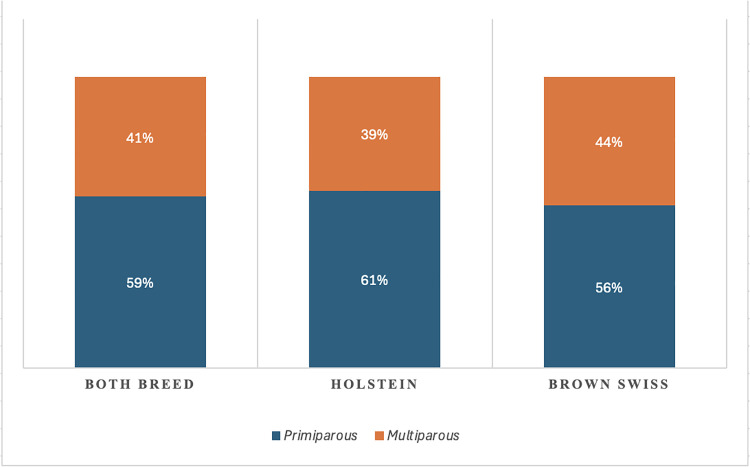
Distribution of cows by parity order (primiparous, n = 1451 vs. multiparous, n = 1009) in the overall population (Both breeds n = 2460), and separately for Holstein (n = 1386) and Brown Swiss (n = 1074). Percentages are indicated within each bar.

The distribution of animals according to days post-AI and DIM is reported in Fig. 2. For days post-AI, the overall median was 55 days, with values of 57.5 days in Brown Swiss and 54 days in Holstein cows. The corresponding means were 65.3 ± 33.9, 68.2 ± 35.6, and 62.8 ± 32.3 days, respectively. In all groups, the distribution ranged from 28 to 200 days. Regarding DIM, the overall median was 210 days, with a mean of 221.6 ± 88.0. Brown Swiss cows exhibited slightly higher DIM values, with a median of 220 and a mean of 228.0 ± 88.0, whereas Holstein cows showed a median of 201 days and a mean of 216.4 ± 87.6. Across both breeds, DIM values spanned from 80 to 500 days. The joint distribution of days post-AI and DIM is depicted in Fig. 3, where the boxplots illustrate the broader variability of DIM compared with days post-AI across both breeds. These findings indicate that the dataset encompassed cows at heterogeneous reproductive and lactational stages, with comparable distributions between Holstein and Brown Swiss breeds. Regarding parity order, 59% of cows in the overall dataset were primiparous and 41% multiparous (Fig. 4). Stratification by breed revealed that Holstein cows were predominantly primiparous (61%), while multiparous accounted for 39%. In contrast, Brown Swiss showed a more balanced distribution, with 56% primiparous and 44% multiparous.

**Fig. 5:**
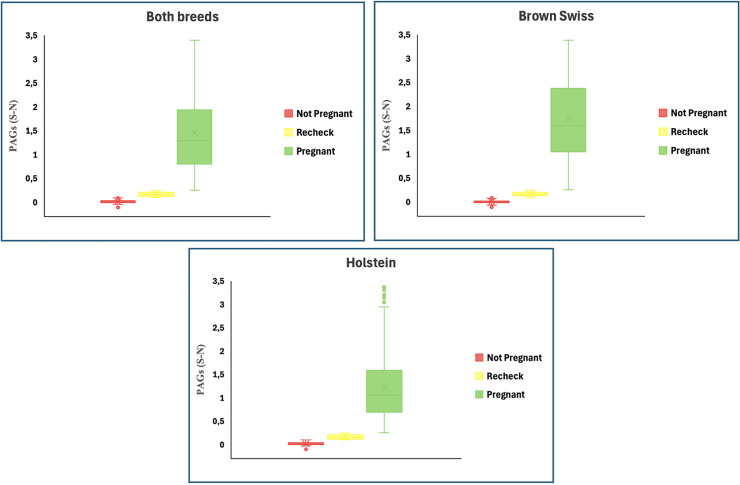
Distribution of PAGs (expressed as S-N values) across the three diagnostic categories (Pregnant (n = 1750), Recheck (n = 142), and Not pregnant (n = 568) in the overall dataset (Both breed, n = 2460) and separately for Brown Swiss (n = 1074) and Holstein cows (n = 1386). Boxplots display the median (horizontal line in the bloxplot) and the mean value (cross in the boxplot).

The distribution of milk PAGs (calculated as the optical density and expressed as S-N values) across the three different database is shown in Fig. 5. In the overall population, cows classified as Not pregnant had PAGs concentrations close to baseline, with average values (± s.e.) of 0.015 ± 0.035 and a median of 0.010. Animals in the Recheck group showed slightly higher concentrations, with mean values (± s.e.) of 0.167 ± 0.047 and a median of 0.158. By contrast, the Pregnant group was characterized by substantially higher concentrations, with an overall mean (± s.e.) of 1.455 ± 0.831 and a median of 1.29. When the data were stratified by breed, some differences emerged in the absolute values.

In Brown Swiss cows, Not pregnant animals showed extremely low concentrations, with mean (± s.e.) and median values of 0.007 ± 0.033 and 0.003, respectively. The Recheck group maintained average concentrations of 0.168 ± 0.043, with a median of 0.161, while the Pregnant group reached the highest levels, with a mean (± s.e.) of 1.75 ± 0.879 and a median of 1.60, indicating overall higher PAGs values in comparison with Holsteins.

In Holstein cows, baseline concentrations in the Not pregnant group were slightly higher than those of Brown Swiss, with a mean value (± s.e.) of 0.024 ± 0.031 and a median of 0.018. The Recheck animals again showed mean concentrations of 0.166 ± 0.049 and a median of 0.155. In the Pregnant group, the concentrations reached average values of 1.23 ± 0.715, with a median of 1.06, which were consistently lower than those observed in Pregnant Brown Swiss group.

### 3.2 PAGs and pregnancy outcome

**Fig. 6:**
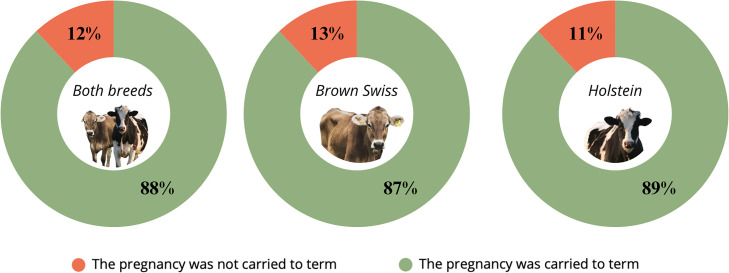
Pregnancy outcomes of cows classified as pregnant based on PAGs testing, showing the proportions of animals that subsequently carried the pregnancy to term or experienced pregnancy loss. Data are presented for the overall population (Both breeds, n = 2460) and separately for Brown Swiss (n = 1074) and Holstein cows (n = 1386).

From the evaluation of pregnancy outcomes, it emerged that none of the 142 cows categorized as recheck successfully carried the pregnancy to term. In contrast, among the cows testing positive, 88% carried the pregnancy to term, whereas 12% failed to do so (Fig. 6). These cases of pregnancy loss may be attributable to abortion, embryonic resorption, or culling; however, since such information is not available in the database, the specific cause could not be determined.

**Fig. 7:**
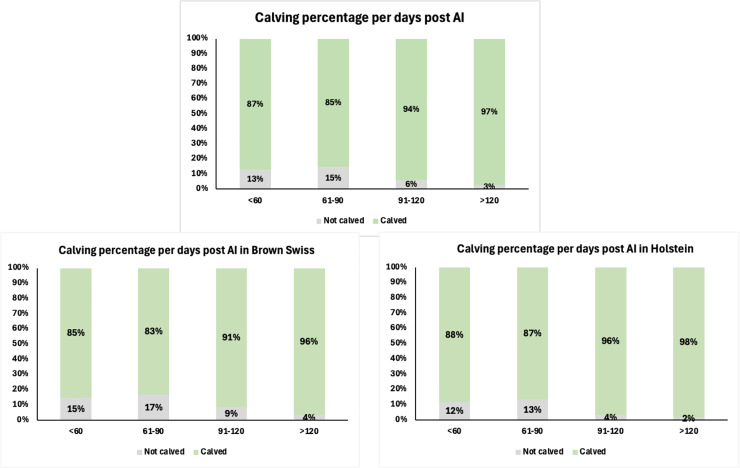
Calving outcomes of cows classified as pregnant by PAGs testing, expressed as the percentage of animals that carried the pregnancy to term according to the interval from artificial insemination (AI) to sampling (<60, 61–90, 91–120, and >120 days post-AI). Results are shown for the overall population (at the top, n = 2460) and stratified by breed, with separate panels for Brown Swiss (bottom left, n = 1074) and Holstein (bottom right, n = 1386).

Animals in which pregnancy did not result in calving were stratified according to the days post-insemination at which pregnancy diagnosis by PAGs was performed, as illustrated in Fig. 7. Pregnancy losses were not evenly distributed across the different post-AI intervals. In the overall population, the proportion of cows that failed to calve was higher when pregnancy was diagnosed before 90 days post-AI, with losses of 13% in animals sampled before 60 days and 15% in those between 61 and 90 days. By contrast, the rate of pregnancy loss decreased substantially in cows sampled later in gestation, with only 6% and 3% failing to calve in the 91–120 day and >120 day categories, respectively.

When stratified by breed, a similar temporal pattern was observed. Brown Swiss cows showed losses of 15% and 17% in the <60 day and 61–90 day groups, respectively, whereas pregnancy loss dropped to 9% and 4% beyond 90 days. In Holsteins, early pregnancy losses were slightly lower, with 12% and 13% in the first two intervals, followed by a marked decline to 4% and 2% in cows sampled after 90 days post-AI.

### 3.3 PAGs and gestational age

**Fig. 8:**
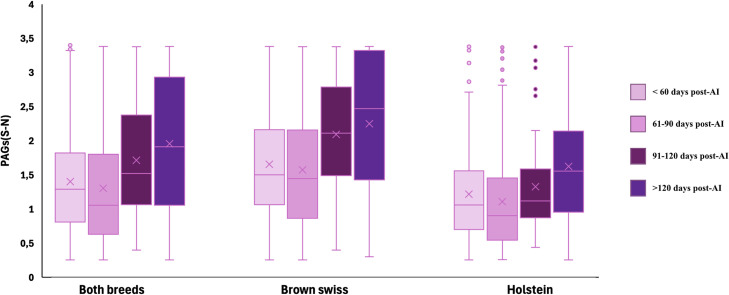
Distribution of PAGs values (S-N) across different gestational intervals in the overall population (Both breeds n = 2460), Brown Swiss (n = 1074), and Holstein cows (n = 1386). Boxplots display the median (horizontal line) and mean (cross).

Milk PAGs concentrations showed significant variations across different stages of gestation, as summarized in Table 1 and illustrated in Fig. 8. In the overall population, PAGs levels decreased significantly between cows pregnant for <60 days and those between 61 and 90 days of gestation. Thereafter, concentrations increased progressively, with statistically significant differences observed when comparing cows pregnant for <90 days with those at 91–120 days and beyond 120 days.

**Table 1:** Mean PAGs (expressed as S–N values) according to days post-AI (<60, 61–90, 91–120, and >120 days) in the overall population (Both breeds, n = 2460) and separately in Holstein (n = 1386) and Brown Swiss cows (n = 1074).

		Days post-AI				SEM^2^	*P*-value
		*<60*	*61–90*	*91–120*	*>120*		
PAGs^1^ (S-N)	***Both breeds***	1.36^Aa^	1.18^B^	1.57^ACb^	1.76^C^	0.06	<0.001
	***Holstein***	1.17^a^	0.98^Abc^	1.21^ad^	1.47^Bc^	0.07	<0.001
	***Brown Swiss***	1.59^A^	1.49^A^	2.01^B^	1.98^B^	0.10	<0.001

In line: A,B,C: p < 0,01; a,b,c,d: p < 0,01.

1 PAGs: Pregnancy Associated Glycoproteins;.

2 SEM: standard error of the mean.

**Fig. 9:**
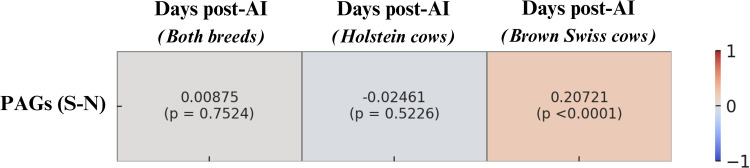
Heatmap of Pearson correlation coefficients between PAGs (S–N) concentrations and days post-artificial insemination (AI) in pregnant cows, considering the entire population (Both breeds n = 2460), Holstein cows (n = 1386), and Brown Swiss cows (n = 1074). Each cell reports the correlation coefficient and the corresponding statistical significance (p-value).

When stratified by breed, Italian Holstein cows displayed a trend closely resembling that of the overall population, with an initial decline followed by a progressive rise in milk PAGs concentrations as gestation advanced. In contrast, Italian Brown Swiss cows exhibited a different pattern. In this breed, no significant differences were detected between cows pregnant for <60 days and those between 61 and 90 days, nor between animals at 91–120 days and those beyond 120 days of gestation. However, Brown Swiss cows did show significantly higher concentrations in late gestation, with differences evident between animals sampled before 90 days and those sampled after 120 days. Moreover, the analysis of correlation coefficients between milk PAGs concentrations and days of gestation revealed a statistically significant positive correlation only in Brown Swiss cows (Fig. 9).

### 3.4 PAGs and DIM

**Fig. 10:**
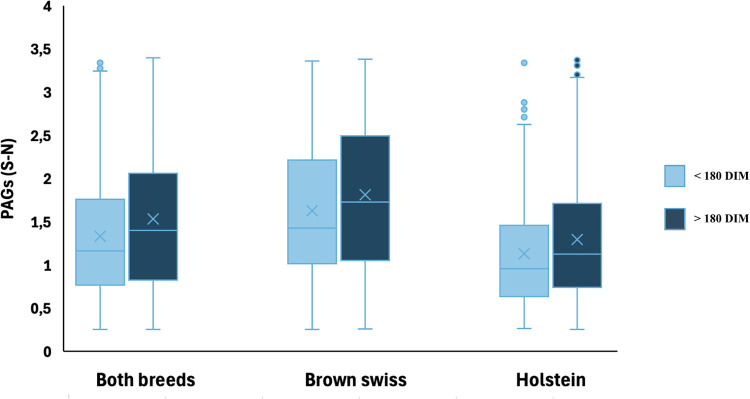
Distribution of PAGs values (S-N) in cows with <180 DIM and >180 DIM. Data are shown for the overall population (Both breeds n = 2460) and separately for Brown Swiss (n = 1074) and Holstein cows (n = 1386). Boxplots indicate the median (horizontal line) and the mean (cross).

No significant differences were detected in milk PAGs concentrations between cows with <180 DIM and those with >180 DIM. This result, consistent across the entire dataset as well as when considering Holstein and Brown Swiss cows separately, is graphically illustrated in Fig. 10.

**Fig. 11:**
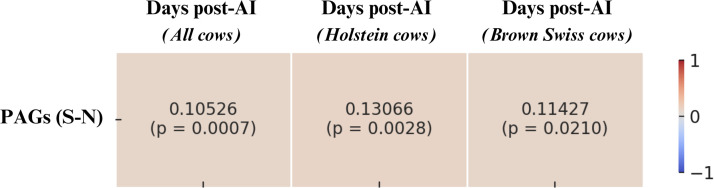
Heatmap of Pearson correlation coefficients between PAGs (S–N) concentrations and days in milk (DIM) in pregnant cows, considering the entire population (Both breeds n = 2460), Holstein cows (n = 1386), and Brown Swiss cows (n = 1074). Each cell reports the correlation coefficient and the corresponding statistical significance (p-value).

The Pearson test revealed a positive correlation between milk PAGs concentrations and DIM in both Italian Holstein and Brown Swiss cows. The corresponding correlation coefficients are presented in Fig. 11.

### 3.5 PAGs and parity

In relation to parity, milk PAGs concentrations differed significantly between primiparous and multiparous cows, as summarized in Table 2. When breeds were analyzed separately, no significant differences were detected in Italian Holstein cows, whereas in Brown Swiss cows multiparous animals exhibited higher PAGs concentrations compared with primiparous cows.

**Table 2:** Mean PAGs (expressed as S–N values) in primiparous (n = 1451) and multiparous (n = 1009) cows. Data are reported for the overall population (Both breeds, n = 2460) and separately for Holstein(n = 1386) and Brown Swiss cows (n = 1074).

		Parity		SEM^2^	*P*-value
		Primiparous	Multiparous		
PAGs^1^ (S-N)	***Both breeds***	1.41^a^	1.53^b^	0.04	0.026
	***Holstein***	1.19	1.33	0.06	0.104
	***Brown Swiss***	1.68^a^	1.95^b^	0.08	0.037

In line: a,b: p < 0.05.

1 PAGs: Pregnancy Associated Glycoproteins;.

2 SEM: standard error of the mean.

### 3.6 PAGs and milk parameters

**Fig. 12:**
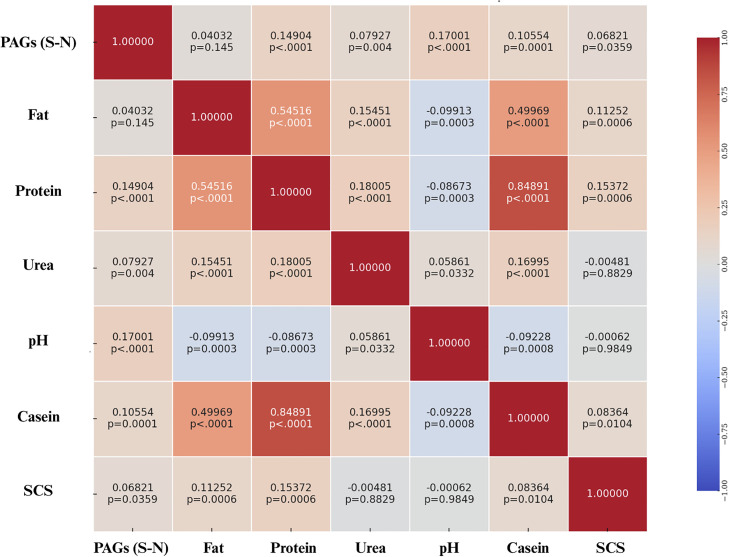
Heatmap of Pearson correlation coefficients between PAGs (S–N) and the main physicochemical milk parameters (fat, protein, urea, pH, casein, and SCS), calculated using the entire dataset. Each cell displays the correlation coefficient and its corresponding statistical significance (p-value). Correlation values are color-coded from –1 (blue) to +1 (red), as indicated by the side bar.

**Fig. 13:**
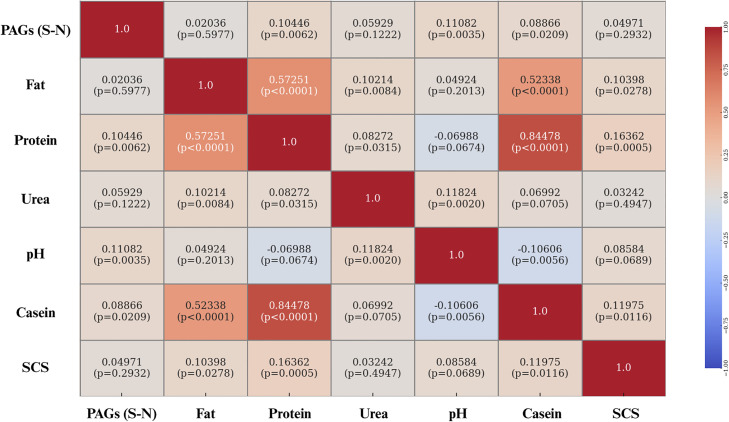
Heatmap of Pearson correlation coefficients between PAGs (S–N) and the main physicochemical milk parameters (fat, protein, urea, pH, casein, and SCS) in Holstein cows. Each cell displays the correlation coefficient and its corresponding statistical significance (p-value). Correlation values are color-coded from –1 (blue) to +1 (red), as indicated by the side bar.

**Fig. 14:**
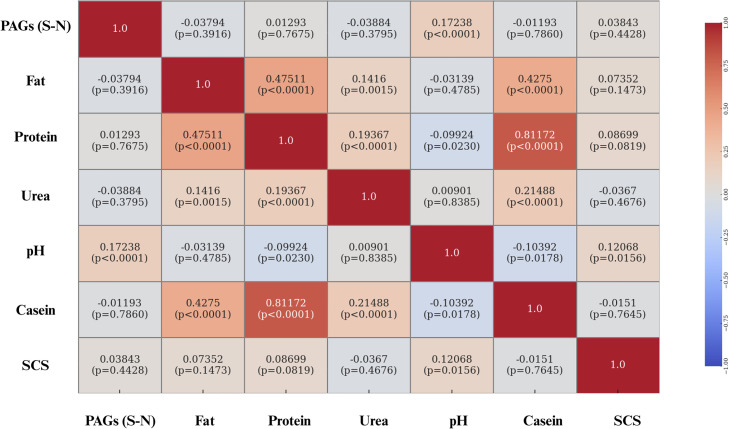
Heatmap of Pearson correlation coefficients between PAGs (S–N) and the main physicochemical milk parameters (fat, protein, urea, pH, casein, and SCS) in Brown Swiss cows. Each cell displays the correlation coefficient and its corresponding statistical significance (p-value). Correlation values are color-coded from –1 (blue) to +1 (red), as indicated by the side bar.

Pearson correlation coefficients between PAGs (S-N) levels in milk and key milk parameters such as fat, protein, urea, pH, casein, and SCS are presented in the heatmap below (Fig. 12). Weak positive correlations were observed between PAGs and protein, urea, casein contents, and milk pH. When considering only Holstein cows, a weak positive correlation was found between PAGs levels and protein content, urea, and pH, as shown in Fig. 13. In contrast, for Brown Swiss cows, weak correlations were observed between PAGs levels and both milk pH and casein content. The correlation coefficients are shown in Fig. 14.

## 4 Discussion

Analysis of the database revealed that the study population was evenly distributed with respect to reproductive parameters, lactation stage, and parity, thereby minimizing potential confounding effects related to herd structure. This homogeneity strengthens the robustness of subsequent comparisons and supports the reliability of the results obtained.

Milk PAGs values (expressed as S-N) confirmed a clear separation among the three diagnostic categories (Pregnant, Recheck, and Not pregnant). Moreover, breed-related differences emerged in the absolute concentrations of PAGs. Brown Swiss cows tended to exhibit higher PAGs concentrations than Holsteins in both the Pregnant and Not Pregnant groups. These findings may reflect subtle breed-specific differences in placental function or in the transfer of PAGs into milk. In particular, breed-related differences in the expansion of placental development and in cotyledonary surface area have been reported in cattle (Van Eetvelde et al., 2016). Although direct evidence comparing Brown Swiss and Holstein cows is lacking, it is reasonable to hypothesize that the substantial metabolic differences between these two breeds may also influence placental morphology and function (Catellani et al., 2023; El-Tarabany et al., 2017). Such variations could affect not only the growth dynamics of the placenta but also its endocrine activity and capacity for nutrient exchange, ultimately contributing to the observed differences in PAGs concentrations (Wallace et al., 2015).

Nonetheless, one consistent feature across all analyses was the distinct gap between animals classified as positive (Pregnant) and those requiring further evaluation (Recheck). This wide separation underscores the diagnostic robustness of PAGs testing, as even in the presence of breed-related variability, the discrimination between confirmed pregnancies and uncertain cases remains clearly defined. This interpretation aligns with the established biology and diagnostic performance of PAGs in milk and plasma (Sousa et al., 2006; Ricci et al., 2015; Mayo et al., 2016).

From the results obtained, it emerged that among the animals diagnosed as “pregnant” based on PAGs concentrations, approximately 12% did not carry the pregnancy to term. This proportion is consistent with the values reported in the literature when considering together the average culling rate of pregnant cows and the mean incidence of embryonic resorptions and abortions (Pinedo et al., 2010; Andrade et al., 2024; Mee, 2023). Although the present database did not allow us to discriminate between these causes, the observed rate of pregnancy loss can reasonably be attributed to one or more of these events (Mee, 2023; Andrade & Simões, 2024).This finding highlights the need for periodic monitoring of dairy cows during gestation through rectal palpation and ultrasonography. Indeed, although PAGs quantification represents a sensitive and practical method for early pregnancy diagnosis, it cannot be considered exhaustive unless integrated with direct clinical evaluation of the animals. Evidence from longitudinal studies shows that reduced circulating or milk PAGs precede or accompany pregnancy loss, which reinforces the value of confirmatory clinical follow-up (Pohler et al., 2016; Barbato et al., 2022; Holton et al., 2022).

Regarding the animals classified as “recheck,” none carried the pregnancy to term. This evidence can be explained by the decline in PAGs concentrations that occurs following abortion or embryonic resorption (Wallace et al., 2015; Barbato et al., 2022; Ask-Gullstrand et al., 2023). During this phase, PAGs values initially fall within the “recheck” range before decreasing to undetectable levels. Therefore, the 142 cows diagnosed as “recheck” likely represented cases in which abortion or resorption had already occurred, resulting in pregnancy loss. This suggests that the “recheck” range may serve as an early indicator of abortive or resorptive events, in which PAGs concentrations are already in decline (Barbato et al., 2016; Ask-Gullstrand et al., 2023). Based on this evidence, cows classified as “recheck” should be specifically flagged within herd management protocols. In particular, these animals could be brought to the attention of the veterinarian responsible for reproductive management for further clinical evaluation. This strategy may facilitate the early identification of cows at risk of pregnancy loss and improve decision-making at the farm level.

Analysis of PAGs dynamics across gestational stages showed a decrease in milk PAGs concentrations between animals pregnant for < 60 days and those pregnant between 61 and 90 days. This decline was followed by a progressive increase in PAGs concentrations up to day 180 of gestation, which represented the temporal limit considered in this study. The reduction in mean PAGs levels in milk between 60 and 90 days of gestation may be due to the presence of a PAGs peak in milk around day 32 of gestation, as previously reported (Ricci et al., 2015), as well as to the higher incidence of suspected abortions and resorptions observed in this group. These events, by lowering PAGs concentrations in milk, may have contributed to the reduced mean values observed when pregnancy diagnosis was performed between 61 and 90 days of gestation. The described early peak, subsequent nadir (≈ 46–67 days post-AI), and later rebound in PAGs are well-documented, supporting the biological plausibility of our pattern (Ricci et al., 2015). This pattern, derived from the entire dataset, was similar to that observed when only Holstein cows were considered. Conversely, in Brown Swiss cows, milk PAGs concentrations remained relatively stable up to approximately 90 days of gestation, without significant fluctuations during this interval. A clear increase in PAGs levels was only observed after day 90. Therefore, unlike in Holsteins, no appreciable differences in milk PAGs concentrations were detected between the period prior to 60 days and that between 60 and 90 days of gestation in Brown Swiss cows. This trend may reflect a distinct temporal pattern of PAGs secretion or transfer into milk, potentially associated with physiological or placental characteristics specific to the breed; population-scale datasets demonstrate that PAGs signal dynamics and pregnancy-maintenance traits vary among breeds and genetic backgrounds (Ask-Gullstrand et al., 2023). In addition, it cannot be excluded that the observed PAGs profiles are partially influenced by the temporal expression patterns of individual PAGs isoforms (Green et al., 2000). Different PAGs family members are indeed known to be expressed preferentially at specific gestational age (Green et al., 2000); therefore, at certain gestational windows, PAGs isoforms that are effectively recognized by the ELISA assay may be more abundantly expressed than at others. This differential temporal expression could contribute to subtle variations in measured PAGs concentrations across gestation, independent of overall placental mass or function.

Moreover, correlation analysis revealed a weak positive relationship between milk PAGs concentrations and days of gestation only in Brown Swiss cows, whereas no correlation was observed in Holsteins. This difference may be attributed to the distinct patterns of PAGs dynamics in milk between the two breeds: in Brown Swiss cows, concentrations remain stable until about 90 days of gestation and then increase, without the initial decline followed by a subsequent rise observed in Holsteins. The biological basis for such variability is consistent with reports that circulating/milk PAG profiles are influenced by placental development and maternal factors (Patel et al., 1997).

Regarding the effect of DIM on PAGs concentrations in milk, no significant correlations were detected in either Holstein or Brown Swiss cows. This indicates that PAGs, primarily reflecting trophoblastic activity, are more strongly influenced by the stage of pregnancy than by the phase of lactation, in agreement with the physiology and diagnostic use of the PAG family (Sousa et al., 2006).

Additionally, this study found higher milk PAGs concentrations in multiparous cows compared with primiparous ones. This finding contrasts with previous reports in which higher PAGs levels were observed in primiparous animals. A possible explanation may lie in the tendency of multiparous cows to develop larger placentas with a greater number of trophoblastic cells, which are responsible for PAGs synthesis (Redifer et al., 2021). Furthermore, as parity increases, uterine and placental blood flow also rise, potentially facilitating the transfer of PAGs from systemic circulation into milk and thereby contributing to the higher concentrations observed in multiparous cows (Panarace et al., 2006; Redifer et al., 2024). Notably, earlier Holstein studies reported higher milk and plasma PAGs in primiparous cows (Ricci et al., 2015; Fodor et al., 2019); differences in breed composition, physiological status, sampling windows, and analytical platforms may contribute to these discrepancies (Panarace et al., 2006; Redifer et al., 2024).

Regarding the interactions between milk parameters and PAGs concentrations, weak positive correlations were identified with three variables: protein content, urea concentration, and casein percentage. The correlation between PAGs and protein content may be partly explained by the biochemical nature of PAGs, which are glycoproteins (Touzard et al., 2013; Gajewski et al., 2014). Consequently, an increase in their concentration in milk may directly contribute to an increase in total protein content, justifying the positive correlation observed. An additional explanation may relate to the way milk proteins are expressed, i.e., as concentrations per unit volume. As pregnancy advances, DIM also increase, while milk yield tends to decline; hence, concentration effects can raise protein and casein percentages independently of absolute secretion (Pollott, 2000; Marino et al., 2021). In this context, the relative increase in protein content may coincide with the rise in PAGs levels, giving rise to the positive correlation between the two parameters (Sousa et al., 2006; Gajewski et al., 2009).

The positive correlation observed between casein percentage and PAGs concentrations in milk can be interpreted similarly. Caseins, like total protein, are expressed as concentration per unit volume rather than absolute quantity; as milk volume decreases later in lactation, casein percentages may increase because of reduced dilution (Marino et al., 2021). Since PAGs levels also increase with advancing gestation, it is plausible that the positive correlation between PAGs and casein reflects an indirect effect linked to gestational physiology rather than a direct interaction between the two variables (Marino et al., 2021).

Finally, the positive correlation between PAGs concentrations and milk urea levels may be interpreted as an indirect effect of advancing gestation and DIM. Indeed, higher PAGs levels are generally found in later stages of pregnancy, during which nitrogen-use efficiency is often reduced; higher systemic surplus nitrogen is converted to urea and appears in both blood and milk (Jonker et al., 1998; Bobbo et al., 2020; Tavernier et al., 2023). Breed differences in urea handling (with Brown Swiss often exhibiting higher MUN than Holsteins) could further modulate this association in mixed-breed datasets (Kessler et al., 2024).

Some limitations of this study should be acknowledged. Firstly, due to its retrospective design, it was not possible to confirm pregnancy status via ultrasonography at the time of PAGs measurement. However, it is noteworthy that 88% of the cows diagnosed as pregnant subsequently carried the gestation to term, supporting the reliability of the test and suggesting that false positive results were minimal in this population. For the remaining 12% of cows that did not complete the pregnancy, the lack of ultrasonographic confirmation represents a relevant limitation, as it was not possible to determine whether these animals had already experienced embryonic loss at the time of PAGs testing or whether pregnancy loss occurred subsequently. Furthermore, detailed information on pregnancy losses (embryonic resorption or abortion) was unavailable. Consequently, the association between declining PAGs concentrations and pregnancy loss cannot be demonstrated directly, but can only be inferred from the subsequent failure of these animals to carry the pregnancy to term. Additionally, the retrospective nature of the study did not allow full control over sampling conditions, although these are generally standardized within routine milk recording procedures. Despite these limitations, this study has several important strengths. The large sample size (n = 2460) provides robust statistical power and enhances the reliability of the results. Furthermore, including multiple variables enabled a comprehensive evaluation of factors influencing PAGs concentrations. Finally, using field data derived from routine herd management practices ensures the findings are highly representative of real-world conditions, making them more applicable to dairy herd reproductive management in practice.

## 5 Conclusion

In conclusion, this study suggests that quantification of PAGs in milk represents a potentially useful and practical tool for early pregnancy assessment in dairy cows, providing a clear distinction among pregnant, recheck, and not pregnant categories across the population. Absolute PAGs concentration, showed breed-specific differences, generally higher in Brown Swiss than in Holstein and varied significantly according to gestational age, whereas meaningful association was observed with DIM, indicating that lactation stage exerts a minor influence compared with pregnancy stage. Approximately 12% of cows testing positive did not carry the pregnancy to term, underscoring that PAGs testing should not be used as a stand-alone diagnostic. Accordingly, pregnancy diagnosis based on PAGs can be considered a valuable tool to be integrated into herd reproductive management programs. This method is relatively inexpensive, easy to implement and does not require invasive or repeated animal handling. Its implementation could allow veterinarians to focus clinical examinations on non-pregnant and recheck cows, thereby optimizing time management and increasing the overall efficiency of reproductive monitoring at the herd level.

## Ethical statement

Not applicable.

## CRediT authorship contribution statement

**G. Bramante:** Writing – original draft, Visualization, Investigation, Data curation. **L. Forte:** Writing – original draft, Visualization, Software, Methodology, Formal analysis. **O.M. Andriulo:** Investigation, Data curation. **A. Carbonari:** Investigation, Data curation. **A. Maggiolino:** Writing – original draft, Conceptualization. **P. De Palo:** Writing – review & editing. **A. D’Onghia:** Methodology, Data curation. **G.E. Dahl:** Writing – review & editing. **V. Cicirelli:** Writing – review & editing. **A. Rizzo:** Supervision, Resources, Project administration, Funding acquisition.

## Declaration of competing interest

The authors declare that there are no conflicts of interest regarding the publication of this work.
